# Recent Advances and Prospects in Design of Hydrogen Permeation Barrier Materials for Energy Applications—A Review

**DOI:** 10.3390/molecules27196528

**Published:** 2022-10-02

**Authors:** Ewa C. E. Rönnebro, Robert L. Oelrich, Robert O. Gates

**Affiliations:** Pacific Northwest National Laboratory, Richland, WA 99352, USA

**Keywords:** hydrogen permeation, hydrogen absorption, barrier coatings, hydrogen pick-up, tritium, renewable energy, nuclear reactor, fusion reactor, radiation resistance, coating manufacturing, tritium permeation barrier, permeation reduction factor

## Abstract

The hydrogen infrastructure involves hydrogen production, storage and delivery for utilization with clean energy applications. Hydrogen ingress into structural materials can be detrimental due to corrosion and embrittlement. To enable safe operation in applications that need protection from hydrogen isotopes, this review article summarizes most recent advances in materials design and performance characterization of barrier coatings to prevent hydrogen isotopes’ absorption ingress and permeation. Barriers are crucial to prevent hydride formation and unwanted hydrogen effects to increase safety, materials’ lifetime and reduce cost for applications within nuclear and renewable energy. The coating may be applied on a material that requires protection from hydrogen pick-up, transport and hydride formation in hydrogen storage containers, in pipelines, spent nuclear fuel storage or in nuclear reactors. While existing, commercial coatings that have been much in use may be satisfactory for various applications, it is desirable to evaluate whether alternative coating concepts can provide a greater resistance to hydrogen isotope permeation along with other improved properties, such as mechanical strength and thermal resistance. The information presented here is focusing on recent findings within the past 5–7 years of promising hydrogen barriers including oxides, nitrides, carbon, carbide, MAX-phases and metals and their mechanical strength, hydrogen pick-up, radiation resistance and coating manufacturing techniques. A brief introduction to hydrogen permeation is provided. Knowledge gaps were identified to provide guidance for material’s research prospects.

## 1. Introduction

### 1.1. Hydrogen Barriers

The hydrogen infrastructure includes hydrogen production, storage and delivery. To store hydrogen in materials with high gravimetric and volumetric energy density, a variety of different classes of materials have been explored extensively in the past 20 years. If, however using containers to store hydrogen gas, materials that do not pick-up hydrogen is needed. To further reduce hydrogen pick-up and permeation in container materials, a hydrogen barrier can be applied as a coating on the substrate materials.

Hydrogen barriers are crucial to enable safe energy solutions in support of a clean energy infrastructure and for safety of nuclear reactors such as fusion reactors, development of future Gen IV nuclear reactors as well as to contain spent fuel [1,2,3]. Some of the Gen IV reactors will be used for hydrogen production and some use high-temperature salt-coolants [4]. With an excellent barrier, accidents could be avoided or reduced in severity by preventing tank, structural or pipe-line material failure and help mitigate the consequences of Zirconium-water reactions in nuclear reactor incidents such as the one in Fukushima in 2011 which spurred an increase in materials research. By preventing ingress of hydrogen isotopes, the material will exhibit longer lifetime and avoid costly replacements of parts due to embrittlement. For nuclear applications, many of these hydrogen barriers exhibit excellent permeation-resistant behavior in the laboratory but fail when used in a radiation environment. Irradiation and implantation can affect the transport of hydrogen isotopes in materials. In addition to resistance to hydrogen absorption and permeation, the coatings must be reasonably ductile and have good strength to preclude cracking during fabrication and service.

Various materials which could serve as effective hydrogen barriers have been identified and some are commercially available. Their function for a desired application depends on various factors including temperature, pressure, microstructure, coating thickness, compatibility with the substrate and sometimes compatibility with chemical reactions. Therefore, the barrier must be carefully selected per application along with appropriate manufacturing method. Among classes of hydrogen permeation barriers that have been explored are oxides, nitrides, carbides, carbon, metals and intermetallics. Although metals such as tungsten and gold possess permeabilities low enough to permit them to be considered as hydrogen permeation barriers, most of the recent work has been focused on ceramic-based barriers. 

The hydrogen barrier is typically applied as a thin coating/film onto a substrate material, although the container itself can be the hydrogen barrier [3]. With the emerging of nanoscience and computational science, several novel approaches and strategies have been applied in recent years to design better hydrogen permeation barriers that can also meet other requirements. The following minimum criteria needs to be considered for the function of a hydrogen barrier:

▪ Reduced hydrogen pick-up to avoid hydrogen transport and formation of hydrides and hydrogen effects
▪ Show mechanical strength
  o Coating needs to maintain integrity and to not weaken strength of cladding
▪ Reduced embrittlement due to hydride formation during service
▪ Reduced thermal stress or thermal expansion between cladding and coating
▪ Corrosion resistance (in water and/or oxygen)
▪ Manufacturing feasibility

In addition, the following criteria are important in nuclear applications:

▪ Reduce hydrogen isotope permeation into the reactor coolant system (RCS)
▪ Reduce hydrogen isotope ingress into nuclear reactor’s structural materials
▪ High-temperature resistance up to loss-of-coolant accident (LOCA) conditions (≥1200 °C)
▪ Neutron transparency
▪ Long term endurance in the radiation environment of a nuclear reactor

With this review article, we have summarized advanced coatings and prospects to prevent hydride formation and hydrogen effects due to hydrogen (H^1^, H^2^, H^3^) pick-up and transportation. When using ‘hydrogen’, we include all isotopes (protium, deuterium and tritium). Research in this area has intensified in the past decade due to the increased investment in development of future nuclear reactors and a hydrogen infrastructure. Most of the cited literature is from within 5–7 years of publication date and represents promising approaches to further advance hydrogen barriers including materials design, nanoengineering and computational science. The hydrogen barrier coating may be applied to a container, such as FeCrAl, zirconium, CrN or SiC, with the purpose to avoid safety concerns due to hydrogen effects that can reduce materials integrity. Or, the container itself can be the hydrogen barrier. For nuclear applications, neutron transparency and minimal impact on the neutron economy also need to be taken in consideration along with radiation endurance. With the understanding that barriers work by limiting the area available for the gas to contact the underlying metal surface, and possibly by creating low enough permeation for recombination to occur and even further reduce the permeation, the questions are how radiation affects this process and whether it enhances permeation which could be detrimental if causing embrittlement in a nuclear reactor and further studies are much needed.

The key challenge is to identify a material that is an excellent hydrogen barrier but not brittle and with mechanical strength. Another challenge is fabrication of the coating without creating cracks or thermal stresses during welding and joining. 

We start out discussing the mechanism for hydrogen permeation, how to measure permeation according to ISO and ASTM standards, and coating/thin film preparation methods. The following sections summarize recent findings for various coatings, i.e., oxides, nitrides, carbon materials, MAX phases, metals and intermetallic alloys and knowledge gaps are identified with a focus on hydrogen barriers for nuclear reactor related applications. To further advance these materials towards commercial use, these selected barriers should be subjected to studies to evaluate materials’ performance related to atomic structure and chemical composition, including mechanical strength, hydrogen pick-up resistance, radiation resistance, corrosion and coating preparation techniques that are feasible for manufacturing.

### 1.2. Mechanism for Hydrogen Permeation

**Figure 1:**
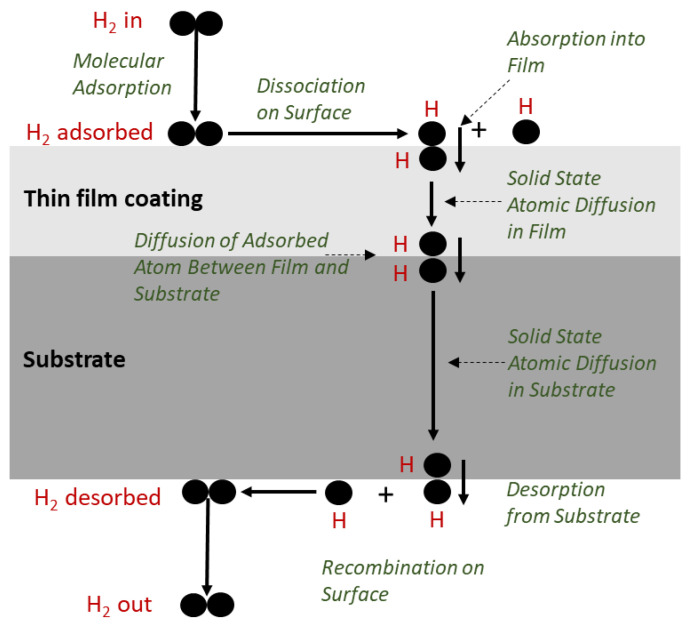
Illustration of hydrogen permeation in a thin film coated substrate; dissociation, adsorption on surface, diffusion in thin film coating, diffusion in substrate, recombination and desorption.

The permeation of hydrogen isotope through solid materials is accepted to proceed via adsorption, dissociation, diffusion, and recombination coupled with desorption. An effective barrier would prevent (or at least significantly reduce) ingress followed by diffusion into film and further into the substrate. Figure 1 shows a schematic illustration of hydrogen permeation in a coated sample if the permeation proceeded all the way through the substrate to desorption and recombination. A hydrogen molecule (H_2_) is adsorbed on the surface of the film and disassociates to hydrogen atoms (H). The adsorbed hydrogen atoms diffuse into the film from the film surface (absorption) and move toward the interface with the substrate. The hydrogen pick-up barrier should effectively prevent hydrogen atoms to diffuse into the film and further into the substrate. Hydrogen atoms can diffuse into the substrate from the interface of the film to the non-coated side of the substrate where the hydrogen atoms form hydrogen molecules and then desorption occurs. When performing a permeation measurement per an ISO procedure, the hydrogen isotope is entering on the coated side and permeates through the substrate to be measured on the other side as discussed below in Section 1.3.

We will briefly discuss the basic equations related to permeation. Permeability of hydrogen and its isotopes is the steady state diffusional transport of atoms through a material as illustrated in Figure 1 that supports a differential pressure of the hydrogen isotope. Wipf showed in 2001 that most metals follow an Arrhenius relationship (or va not Hoff plot) for hydrogen isotope diffusion [5]. Wipf focused on crystalline host metals without defects like vacancies, dislocations and grain boundaries or impurity atoms which have an impact on permeability. Other factors that also impact permeation are microstructure and material composition. Lattice defects are potential hydrogen traps and can therefore modify the behavior of hydrogen substantially. Trapping occurs if potential energies for hydrogen atoms are lowered in interstitial sites. This is equivalent with a larger time of residence in the corresponding sites and, therefore, H-diffusivity is reduced. Kirchheim developed in 2001 a general model based on Statistical Mechanics and Random Walk which allows to describe the behavior of hydrogen in disordered systems, i.e., metallic glasses, amorphous silicon, nanocrystalline metals, deformed metals, disordered metallic solutions, and metallic multi layers [6]. The reader is referred to this paper for the mathematical models.

Assuming a clean surface, permeation (Pm) is the product of diffusion (D_0_) and solubility (S_0_) which are temperature dependent, and both follow Arrhenius equation which can be expressed in following equation where E is activation energy (J/mol), H is enthalpy (J/mol), R is the ideal gas constant and T is temperature (K).

(1) $$\mathrm{Pm}=D_{o}e^{-\frac{E}{\mathrm{RT}}}{*S}_{o}e^{-\frac{\Delta H}{\mathrm{RT}}}$$

Diffusion (D) refers to transport of atoms within a single phase. “Permeation” refers to transport of atoms in molecules in a gas phase across a solid phase to another gas phase. Permeation is diffusion combined with chemical reactions that convert molecules to their constituent atoms that then diffuse across a solid and then, by the same chemical reactions in reverse, form molecular species in a gas phase. Diffusion is driven by differences in concentration within a phase. The permeation through a layer of metal is the diffusion rate in the metal driven by concentration differences across it established through Sievert’s Law.

Fick’s first law of diffusion relates the steady state diffusional flux (J) in mol/m^2^s to diffusivity (D) in m^2^/s and the concentration gradient dc/dx across the solid where c is in mol/m^3^ and x is the thickness in meter. It postulates that the flux goes from regions of high concentration to regions of low concentration, with a magnitude that is proportional to the concentration gradient (spatial derivative). The diffusional flux (J) can be expressed as:

J = −D(dc/dx)(2)

The solubility (S) represents equilibrium between the diatomic hydrogen molecule and hydrogen atoms in a metal according to H_2_ (gas) 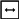 2H (metal). The pressure dependence follows Sievert’s law for the solubility of diatomic gases (hydrogen isotopes) in metals, where c_H_ is concentration of the atomic hydrogen isotope, S is the Sievert’s solubility constant in mol/m^3^Pa^1/2^ and P_H_ is hydrogen pressure (Pa):

c_H_ = S·P_H_^1/2^ (3)

Hydrogen permeation is defined as the transport of hydrogen as dissociated hydrogen atoms and has units of moles of hydrogen gas per square meter per second (mol·m^–2^·sec^−1^), which is the permeation rate. By combining equations 2 and 3, we get a relation that can be expressed as

J = D·S/t (P_high_^1/2^ − P_low_^1/2^) (4)

where J is the permeation rate (mol·m^−2^·sec^−1^), D is the diffusion coefficient of hydrogen in the material, S is solubility coefficient or Sievert’s constant for the material, which determines the hydrogen solubility, and t is the thickness of the solid material. The product of D and S is referred to as Φ, the permeation coefficient or permeability of the material. 

### 1.3. Permeation Measurements—Gas Transmission Rate

The International Organization for Standardization describes ISO 15105 which specifies two methods for determining the gas transmission rate of single-layer plastic film or sheet and multi-layer structures under a differential pressure or equal pressure [7,8]. One method uses a pressure sensor, the other a gas chromatograph, to measure the amount of gas which permeates through a test specimen. The two methods are summarized below.

***Differential pressure method; ISO 15105-1***. A test specimen is placed in a gas transmission cell and forms a sealed barrier between two chambers; a low-pressure (LP) chamber and a high-pressure (HP) chamber that both are evacuated. A gas is introduced into the evacuated HP-chamber and permeates into the LP-chamber. The amount of gas which permeates through the specimen is determined by the increase in pressure on the lower-pressure side or by gas chromatography. The gas transmission rate (GTR) is the volume of gas passing through a plastic material (film/sheet), per unit area and unit time, under unit partial-pressure difference between the two sides of the material. Gas permeability (P) is the volume of gas passing through a plastic material of unit thickness, per unit area and unit time, under unit partial-pressure difference between the two sides of the material.

The gas transmission rate (GTR) and the gas permeability, or coefficient of gas permeability is calculated from Equations below.

If using a gas chromatograph:

GTR = 273 × (V_s_ − V_b_) × k/(22,4·T·A·t·p_h_) (5)

Or if using a pressure sensor:

GTR = V_c_/(R·T · P_h_·A)·dp/dt (6)

where GTR is the gas transmission rate [mol/(m^2^⋅s⋅Pa)]; T is the test temperature (K); t is the time (seconds) during which test gas was collected in the sampling loop; V_s_ is the amount of test gas collected in the sampling loop (liters); V_b_ is the blank reading (liters); V_c_ is the volume of the low-pressure chamber (liters); p_h_ is the pressure in the high-pressure chamber (Pa); A is the transmission area of the specimen (m^2^); k is a conversion factor for converting the sampling loop volume to the total volume of the low pressure chamber; dp/dt is the change in pressure per unit time in the low-pressure chamber (Pa/s); R is the gas constant = 8.31 × 10^3^ (l⋅Pa)/(K⋅mol).

Gas permeability is calculated from GTR and specimen thickness:

P = GTR × d(7)

where P is the gas permeability, or coefficient of gas permeability [mol⋅m/(m^2^⋅s⋅Pa)]; GTR is the gas transmission rate [mol/(m_2_⋅s⋅Pa)]; d is the average thickness of the specimen (meters).

***Equal Pressure Method; ISO 15105-2***. A test specimen is placed in a gas-transmission cell with two chambers forming a sealed barrier in between. One chamber is slowly swept with a carrier gas and the test gas is inserted into the second chamber. Since the partial pressure of the test gas is higher in the second chamber, the test gas permeates through the barrier into the carrier gas in the first chamber. The test gas which permeates through the specimen is carried by the carrier gas to a sensor. Permeability is calculated per above equations.

### 1.4. Permeation Measurements—Electrochemical Technique

ASTM G148 describes a procedure for the evaluation of hydrogen uptake, permeation and transport in metals using an electrochemical technique which was developed by Devanathan and Stachurski [9,10]. This practice describes calculation of an effective diffusivity of hydrogen atoms in a metal and for distinguishing reversible and irreversible trapping. It is based on the steady-state hydrogen flux. In above described solid-gas ISO-15105 permeation measurement, hydrogen atoms are generated by gas adsorption and dissociation on the solid surface. In the electrochemical technique, hydrogen atoms are produced by electrochemical reactions in an electrolyte. The experimental set-up consists of a separate charging and oxidation cell as described in ASTM G148. Specimens may be in the form of plate or pipe. In many cases, the measurements of permeation current versus time are useful in characterizing the severity of hydrogen charging.

The steady state permeation current (J_ss_) in mol/s/cm^2^ gives information on the subsurface concentration of hydrogen atoms at the charging surface as follows:

J_ss_ = J_ss_/(A·F) = D_1·_C_0_/L (8)

where D_1_ is the lattice diffusion coefficient; C_0_ is the sub-surface concentration of atomic hydrogen at the charging side of the specimen (mol/cm^3^); F is Faraday’s constant (9.6485 × 10^4^ coulombs/mol); A is area (cm^2^); L is the specimen thickness (cm). The ASTM G148 procedure provides information related to how to determine D_1_ and analysis of the permeation transient. For all equations related to this method, the reader is referred to ASTM G148. 

### 1.5. Coating Application Methods

**Figure 2:**
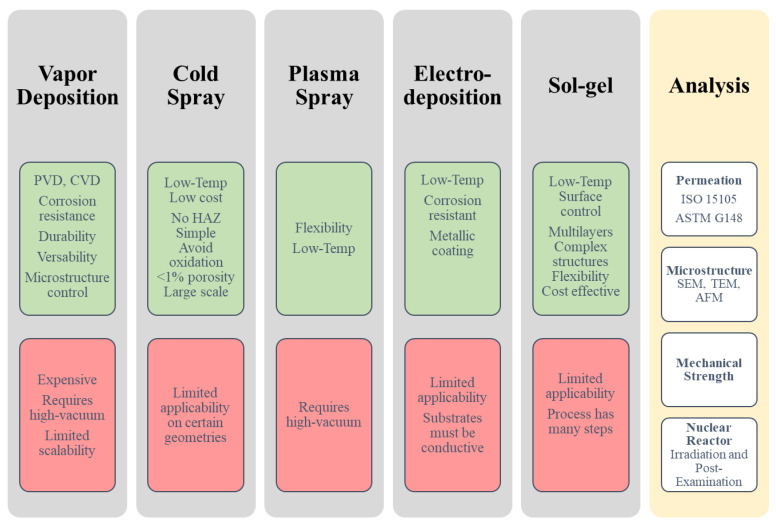
Comparison of five selected Coating Application techniques, pros (green) and cons (red). The right column has basic characterization techniques to analyze hydrogen permeation fabrication applicability, materials integrity, etc.

Selecting the most appropriate coating deposition method can be crucial for the performance of the barrier material. There are several methods to apply coatings including: vapor deposition, sol-gel, spray coating, electrodeposition, pack cementation and hot dipping. A recent extensive review article by Fotovvati et al. from 2019 summarizes coating techniques for surface protection [11] and we will here provide a brief overview to highlight the most common methods, i.e., vapor deposition and a new promising method that industry is adopting, i.e., cold spray. Figure 2 provides a summary of five common techniques and their pros and cons along with important analysis methods. 

There are two main types of vapor deposition processes: chemical vapor deposition (CVD) and physical vapor deposition (PVD). These are atomistic deposition methods that involve vaporization and subsequent deposition of the coating species as a thin film on a substrate. Vapor-deposited coatings are essentially pore-free and dense provided a thick enough layer is applied. This type of coating results in a reduction in the amount of moisture or gas that can penetrate through the film and is therefore considered ideal to reduce corrosion and hydrogen isotope ingress into materials. It has been proposed that coatings applied by PVD could be suited to reduce corrosion in a nuclear environment in present day reactors and in Generation IV type of reactors.

An alternative to PVD and CVD is a relatively new coating process that has been under development since the 80s with recent big strides is Cold Spray (CS) by VRC Metal Systems [12], also referred to as supersonic particle deposition. It is a high-energy solid-state coating and powder consolidation process with the lowest temperatures and highest velocities relative to other thermal spray processes resulting in high strength coatings. The cold spray technique is beneficial when it is necessary to avoid introduction of thermal stress that can occur at high temperatures. It can be used to apply coatings of metals, metal alloys and metal blends for various applications including corrosion-resistant coatings (zinc and aluminum), dimensional restoration and repair (nickel, stainless steel, titanium, and aluminum), wear-resistant coatings (chromium carbide–nickel chromium, tungsten carbide–cobalt, and tungsten copper), electromagnetic interference (EMI) shielding of components and structures, high strength dissimilar material coatings for unique manufacturing solutions, and field repair of components and systems.

Cold spray coatings cause almost no microstructural changes in the powder materials deposited except for extreme plastic deformation, and it does not increase the oxide because the process is so rapid [12]. In fact, the process may reduce or even eliminate the existing oxide layer during cold spray deposition. Other benefits include no “heat-affected zone” (HAZ) due to very low heat input, no real limit on deposition thickness, low porosity below 1%, coating strength above 275 MPa and bond strength above 68 MPa. 

To guarantee fabrication applicability for desired coating on a specific substrate/cladding, several characterization methods are needed including analyzing coating plus cladding/substrate compatibility, materials integrity in applied environment, permeation resistance, mechanical strength. For studying microstructure and surface chemistry, various advanced tools are available, such as SEM, TEM, AFM, XPS, but will not be reviewed here. If the material is aimed for use in nuclear reactors, tests in irradiation are needed to learn irradiation impact on materials integrity and performance. 

## 2. Recent Advances in Hydrogen Permeation Barriers

### 2.1. Oxides

Several oxide coatings have been explored throughout the years, including Al_2_O_3_, Cr_2_O_3_, Er_2_O_3_, ZrO_2_. Recently, focus has been on nano-engineering of oxides to improve properties. Other strategies for materials engineering include structural modifications and using dopants and mixtures. We summarize the most recent findings.

#### 2.1.1. Aluminum Oxide

Aluminum oxide, Al_2_O_3_ (alumina), has been selected for hydrogen permeation barrier on stainless steel and is used in various applications. Alumina exists in many meta-stable polymorphs besides the thermodynamically stable α-Al_2_O_3_ (corundum form) [13]. The commercially available corundum is considered an excellent hydrogen permeation barrier [14,15] with a permeation reduction factor PRF > 1000–10,000, however mechanical and thermal strength needs improvement and during irradiation in a nuclear reactor, the coating does not maintain integrity. Other crystal structures of alumina are not as great hydrogen barriers as the alpha phase. 

Youngman et al. [16] neutron irradiated bulk samples of single crystalline and polycrystalline alpha alumina to doses of 10^26^ n/m^2^ at temperatures of 925 K and 1100 K. The samples were found to swell macroscopically between 3% and 6%, depending on the temperature of irradiation and the form of the material. The damaged microstructures were investigated with transmission electron microscopy (TEM) to understand the origin of the macroscopic swelling and they found that the polycrystalline samples were extensively microcracked, which could be due to anisotropic swelling of the grains which in turn leads to stresses and fracturing at the grain boundaries. Pells reviewed in 1994 known radiation damage mechanisms, including microstructure damage and swelling [17]. It can be noted that experimental in-reactor studies indicate that oxides do not maintain full integrity during prolonged irradiation which leads to radiation damage due to damaged microstructure [15,16]. Therefore, structural modification may be necessary to improve the irradiation tolerance and examples are provided below. 

Morono et al. investigated in 2013 the ionizing radiation induced absorption of hydrogen isotopes in Al_2_O_3_ using thermos-stimulated desorption measurements (TDS) up to 750 °C on both electron irradiated and unirradiated deuterated samples [18]. The TDS results indicate that deuterium desorption temperature increases due to the ionizing radiation that modifies how hydrogen isotopes are trapped within the material, increasing the energy required to desorb. However, the nature of the chemical process is not known. 

In 2014, Greer’s group published a fascinating finding in Science [19]: By creation of ultralight hollow ceramic nanolattices that absorb energy, it can recover after compression. This is a possible path towards enhancing mechanical strength. When compressing lattices by 50% with a lower ratio of 10 nanometers wall thickness to tube diameter leads to deformation that springs back. Although this is a promising result, the synthesis route involves 5 steps which are not likely feasible for low-cost manufacturing. If this phenomenon is to be further explored for a practical application, a new synthesis route needs to be established. 

Another result that points towards improved radiation endurance when using nanolattices was published by García Ferré in 2016 in Scientific Reports [20]. Amorphous/nanocrystalline Al_2_O_3_ thin films were deposited on austenitic steel substrates and irradiated with heavy ions at 600 °C as a surrogate for neutron irradiation. Irradiation induces anamorphous-to-crystalline transformation resulting in a nanograined structure, while continued irradiations induce grain growth. Their findings show promise for use of nanoceramic coatings beyond alumina for in-core, high radiation field components with enhanced corrosion and wear resistance in applications for accident tolerant fuels for advanced light water reactors, fuel cladding for Generation IV systems, and tritium breeding components for fusion tokamaks.

#### 2.1.2. Zirconium Oxide

Zirconium oxide can be used as a hydrogen permeation barrier by naturally forming zirconium oxide on the surface of zirconium or zirconium alloy by corrosion in water or intentional heat-induced oxidation and has mainly been considered for nuclear applications [21]. It has also been used as a debris-resistant feature at the bottom of some fuel rods. Zirconium oxide and yttria stabilized zirconia (YSZ) have been evaluated for hydrogen permeation barriers [22]. 

A coating of zirconium oxide naturally forms on the surface of the zirconium in high-temperature water (such as in a nuclear reactor), and it acts as a protective barrier. If engineered, this layer of oxide can inhibit hydrogen from entering the crystal structure of the metal. A fundamental understanding of the interaction of hydrogen with ZrO_2_ is necessary to allow for designing reliable alloys that can reduce hydrogen entry into the substrate metal. Youssef et al. [23] performed density functional theory (DFT) calculations in 2016 to assess how doping ZrO_2_ with 3*d* transition metals can reduce hydrogen pick-up. They suggested two design strategies that mitigate the ingress of hydrogen into the alloy through this barrier oxide; Either a dopant such as Cr that reduces the solubility of hydrogen in the oxide, or a dopant such as Nb, Ta, W, P, and Mo that speeds up hydrogen evolution at the oxide surface. A future research prospect is experimental validation of the theoretical results. 

Hatano et al. prepared layers of tetragonal ZrO_2_ (180 nm) on the surfaces of ferritic steel substrates by dip coating and electrolytic deposition techniques [24]. These preparation methods were chosen because they can be applied to larger structures. Hydrogen permeation tests were carried out at 300–550  °C and compared with a test of a thinner ZrO_2_ coating (100 nm). The permeation reduction factor (PRF) for the thicker layer was larger by an order of magnitude (ca 1000). The thicker layer results in less defects in the coating which resulted in reduced permeation rate. 

#### 2.1.3. Aluminide + Alumina; FeAl-Al_2_O_3_

Iron aluminides have been among the most studied intermetallics since the 1930s. They have excellent oxidation resistance and their low cost of production, low density, high strength-to-weight ratios, good wear resistance, ease of fabrication make them attractive as a substitute for stainless steel in industrial applications.

Among various developed tritium permeation barrier (TPB) coatings, an aluminide coating typically composed of an inner Fe–Al transition layer with an outer Al_2_O_3_ film has been selected as the first choice of test blanket module (TBM) TPBs by China, Europe, India, and the United States for its high permeation reduction factor (PRF), metallurgical bonding, excellent compatibility, and self-healing. However, in a molten salt reactor (MSR), the aluminide coating is incompatible with the molten salts, and another tritium barrier must be used [25]. 

Oxides, especially Al_2_O_3_ attract much interest as typical candidate TPB materials for their high melting point, chemical stability, low hydrogen solubility and permeability. However, the thermal expansion coefficient shows a large difference between the metal substrate and oxide ceramics, and thus significant thermal mismatch exists, leading to the failure of the coatings when used in applications with wide variations in temperature. The common adopted solution method is to form a functional gradient transition layer between the substrate and the coating, such as the Fe-Al layer.

The preparation process of the aluminide coating generally involves two steps of aluminization and oxidation. Interdiffusion occurs between Al atoms and Fe atoms on the substrate surface to form (Fe, Al) solid solution or Fe-Al intermetallic transition layer in the aluminization step. In the oxidation process, the aluminide layer surface is selectively oxidized to form an Al_2_O_3_ film. The aluminide coating can be prepared by the technique of physical vapor deposition (PVD), chemical vapor deposition (CVD), hot-dipping aluminization (HDA), electro-chemical deposition (ECD), packing cementation (PC), plasma sputtering (PS) and sol-gel, etc. A recent review of aluminide preparation methods was published in 2015 [26].

Yang et al. found in 2016 that the tritium permeability of an γ-Al_2_O_3_/FeAl coated container of 321 stainless steel was reduced by 3 orders of magnitude at 500–700 °C relative to a non-coated container [27], thus the permeation reduction factor (PRF) was in the order of 1000–6000. It is noteworthy that the commercially available α-Al_2_O_3_ is typically used for coatings and has lower permeability, however in Yang’s study, they prepared γ-Al_2_O_3_ using a relatively low temperature route. The Al_2_O_3_/FeAl barrier resists the tritium permeation by the diffusion in the bulk substrate at a limited number of defect sites with an effective area and thickness. The permeation process of the Al_2_O_3_/FeAl barrier can be described by the area defect model and all hydrogen isotopic transport occurs through a limited number of defects with an effective area and thickness.

Xiang et al. investigated how different steel substrates impacts deuterium permeation resistance of aluminide coatings for tritium permeation barrier (TPB) applications [28]. Their conclusion is that the deuterium permeation resistance of the aluminide TPBs depends on the steel substrate composition and microstructure.

The method of doping alloying elements adjust and control the structures and performance of TPB coatings. Prakash showed that increasing carbon and aluminum contents leads to decreased hydrogen diffusivity (increased PRF) [29]. At present, studies on beneficial alloying elements of aluminide coatings are mainly focused on rare earth elements such as Y, Ce and Hf, which can improve the wear, high temperature oxidation and corrosion resistance of aluminide coatings [26]. 

#### 2.1.4. Bipolar Oxide: Chromium Oxide-Aluminum Oxide

Recently Xin et al. showed that a Cr_2_O_3_/Al_2_O_3_ bipolar oxide has better thermal stability than that of Al_2_O_3_/stainless steel and promotes the formation of thermodynamically stable phase of α-Al_2_O_3_ [30]. The bipolar oxide was prepared by first electroplating chromium coating on 316L stainless steel followed by oxidation to form the Cr_2_O_3_ film. Thereafter, high purity metallic Al was sputtered on Cr_2_O_3_ film and then oxidized before finally annealing the bipolar samples. The bipolar oxides are composed of P-type Cr_2_O_3_ semiconducting properties and N-type Al_2_O_3_ and show better hydrogen permeation resistance compared with Cr_2_O_3_ or Al_2_O_3_ coatings. Since aluminum oxide has been shown to have manufacturing feasibility, this bipolar oxide should be similar and therefore this is a new coating recommended to be evaluated with regard to mechanical strength, corrosion resistance, neutron resistance, etc. 

#### 2.1.5. Rare-Earth Oxides

Rare earth oxides have also been explored for hydrogen barrier applications. They have high thermal and mechanical stability in reducing atmosphere. Li et al. [31] studied Er_2_O_3_ coatings on stainless steel in 2017 and showed that nanocrystalline Er_2_O_3_ coating made by sol-gel method can prevent hydrogen isotopes permeation with PRF of 300. In 2016, Mao et al. fabricated high-quality Er_2_O_3_ thin films by ion beam sputter deposition on Si(100) as an epitaxial substrate [32], obtaining very low permeability at 873 K or 4 × 10^−22^ mol Pa^−1/2^ m^−1^ s^−1^ which is several order of magnitude lower than Er_2_O_3_ coatings made by other methods. However, several Er isotopes have relatively strong neutron absorption cross-sections which limits their application in nuclear reactors. 

### 2.2. Nitrides

Nitride based thin films have been shown to reduce hydrogen uptake. The binary nitrides of CrN and ZrN are commercially used as coatings on stainless steels. TiN and CrN have recently been much explored for use in the nuclear industry to prevent corrosion and reduce hydrogen ingress and diffusion into cladding and structural materials. Several groups are working on improving properties for tailored applications by using dopants, multilayers and various fabrication methods to change microstructure features.

#### 2.2.1. Titanium Nitride

Titanium nitride has been proposed as an excellent hydrogen barrier. It has high melting temperature, high hardness, excellent exhibited ion-irradiation tolerance and high thermal conductivity. Recent findings have shown promise in development towards application to prevent corrosion and hydrogen uptake, however further studies of hydrogen permeation behavior is needed. 

Tamura studied hydrogen permeation characteristics of TiN coated stainless steel and measured permeation reduction to be 100 to 5000 times compared to uncoated substrate depending on film’s grain size with the larger grain sizes showing higher hydrogen permeability [33,34]. As is well known, grain boundaries are trap sites for hydrogen, therefore, microcrystalline structures with many grain boundaries are expected to provide effective hydrogen-barrier performance. Their recommendation is to focus future studies to understand the correlation between the hydrogen permeation behavior and microstructure between various film formations.

Zirconium-based alloys such as Zr–1Nb, Zr–2.5Nb are widely used in nuclear industry as cladding materials for nuclear fuel. Kashkarov et al. published a review in 2021 of protective coatings of accident tolerant Zr-based fuel claddings [35]. Hydriding of zirconium alloys leads to degradation of their mechanical properties, hydride cracking and stress corrosion cracking which can be detrimental for structural materials in contact with hydrogen isotopes. Kashkarov et al. researched hydrogenation behavior of titanium nitride (TiN) deposited on Ti-implanted Zr-1Nb alloy [36]. They conducted a comparative analysis on hydrogenation behavior of the Ti-implanted alloy with sputtered and evaporated TiN films. Hydrogenation was performed at 350 °C and 2 atm hydrogen pressure. The lowest hydrogen absorption rate was obtained for Ti-implanted layer with evaporated TiN film and was reduced about 75 times relative to uncoated Zr-1Nb alloy. Morphology of the films impacted hydrogen permeation through TiN films: denser film resulted in lower hydrogen permeation. A dense structure of TiN without voids is necessary along with sufficient grain boundaries that traps hydrogen. Thus, the fabrication of Ti-implanted layer with dense TiN films can be an effective way to protect Zr-1Nb alloy from hydrogen embrittlement. 

Alat et al. studied ceramic multilayered coatings with waterside corrosion resistance in an attempt to develop accident tolerant fuel (ATF) [37]. Hydrogen is evolved during the corrosion reaction which with time will precipitate hydrides in Zr-cladding, causing embrittlement, therefore, it’s important to reduce corrosion. They found that monolithic and multilayer Ti_1−x_Al_x_N (where x ~ 0.54–0.67) and TiN coatings on ZIRLO^®^ substrate by cathodic arc physical vapor deposition (CA-PVD) to improve corrosion resistance. Multilayers with TiN prevents formation of Boehmite and layer architecture optimized to 8 multi layers coating. No crack or delamination/spallation was observed, indicating good corrosion resistance. Only a thin TiN layer is required as a barrier to minimize Al migration and prevent boehmite formation. However, hydrogen permeation was not evaluated and such a study could be of interest for future prospects. 

#### 2.2.2. Chromium Nitride

Chromium nitride is commercially used as a corrosion barrier on stainless steel. Chromium nitride has received a lot of attention in the nuclear industry and nuclear energy world. In 2015, Daub et al. [38] published a study on commercially available coatings of CrN, TiAlN and AlCrN applied by physical vapor deposition to obtain 2–4 μm thick coatings on Zircaloy-4. The corrosion resistance, as well as the impact on hydrogen permeability of these coatings, was investigated. The corrosion resistance was investigated under pure water at 350 °C for 24 h and under CANDU conditions (300 °C and pH 10.5) for 30 d. Limited experiments were also performed in superheated steam at temperatures up to 1100 °C. The CrN-coated samples have shown the best corrosion resistance to date, under pressurized water reactor, CANDU, and superheated steam conditions. Additionally, oxidation of the CrN and TiAlN coatings caused a significant reduction in the hydrogen uptake. 

In 2018, Kashkarov et al. [39] studied CrN_x_ coatings on Inconel 718 superalloy with respect to microstructure, hydrogen permeability and tribology. They found that a mixed cubic CrN and hexagonal Cr_2_N structure has the lowest hydrogen permeability at 873 K (500 °C) and 2 atm hydrogen pressure due to higher packing density of the cubic phase. 

#### 2.2.3. Less Common Nitrides

Matějíček et al. studied “less-common nitrides” for physical vapor deposited coatings of AlCrN, CrN, Cr_2_N, CrWN, WN and ZrN for their suitability as hydrogen permeation barriers [40]. The permeation reduction factor (PRF) ranged from ~10^2^ to ~5×10^3^; the best result being achieved by the ZrN coating and is a promising candidate for future studies. 

Tamura et al. studied a new multi-layered coating of TiAlN/TiMoN [41]. Hydrogen permeabilities of TiAlN-coated, TiN-coated, and TiAlN/TiMoN multi-layered coating samples were measured at 573 K to be 1/100, <1/100 and <1/1000, respectively, relative to the uncoated 316L stainless steel substrate, thus, the new multi-layered coating performs 10 times better as a hydrogen permeation barrier than TiN and TiAlN. 

Houben et al. [42] investigated in 2020 tungsten (W), tungsten nitride (WN) and WN+W layers on Eurofer97 steel substrates. Deuterium permeation measurements at 400 °C showed PRF = 8 for W, PRF = 31 for WN and PRF = 1000 for WNW. The thermal expansion coefficient between W and martensitic steels are different and therefore W coatings cracks during thermal annealing and therefore is not useful as TPB on steels. However, WN and WNW coatings do not crack up and can be used as TPB on steels. 

In 2022, Diaz-Rodriguez et al. [43] studied hydrogen permeation along grain boundaries in nanostructured tungsten deposited on nickel substrate. Their permeation measurements at 520–705 K (247–432 °C) resulted in PRF ~ 4 at all temperatures. The obtained permeability is much higher than reported in the literature for specimens with larger grain size. Their explanation is to be due to grain boundaries perpendicularly oriented relative to surface which facilitates hydrogen migration resulting in higher permeability.

#### 2.2.4. Irradiation Experiments on Nitrides

In-pile testing of CrN, TiAlN and AlCrN coatings on Zircaloy cladding was performed in the Halden Reactor by Van Nieuwenhove et al. [44]. Their conclusions were that TiAlN and AlCrN coatings disappeared but that CrN coating was chemically stable (BWR and PWR). The fact that the TiAlN and AlCrN coatings disappeared is probably related to the fact that the oxide formed on aluminum, namely Al_2_O_3_, dissolved rapidly in BWR or PWR water. Moreover, there was no reduction in coating thickness and CrN coatings reduce hydrogen uptake in zircaloy. Despite accident like conditions, most of the CrN coating was still intact after 150 d. When a crack occurs, oxide forms underneath and the expansion leads to further cracking. Irradiation experiments on ternary and layered nitrides in a hydrogen gas environment to study hydrogen pick-up have not been performed. CrN appears to be a viable hydrogen barrier for use in nuclear reactors. 

***Structural modification*** has been considered as one of the most promising ways to improve irradiation tolerance of transition metal nitride coatings. Numerous structural modification methods have been proposed to enhance the irradiation tolerance of coatings during recent years. Among them, two traditional structure design strategies; multilayer and compositional gradient, have been attracting research interest. A multilayered structure is formed by assembling two different sublayer materials resulting in introducing abundant interlayer interfaces into the coating, which act as sinks to absorb and/or disperse irradiation induced defects. A multilayered coating often shows superior irradiation resistance and mechanical properties. A compositional gradient is produced along the coating thickness direction resulting in continuous change of microstructure and chemical composition, and also absence of interlayer interfaces. A compositionally graded coating has good mechanical reliability; high adhesion strength and thermal shock resistance. It is proposed that a coating system of *hybrid architecture* of multilayered and compositionally graded structures is a new direction to enhance irradiation resistance.

### 2.3. Carbon Materials

Carbon materials have been explored for use as hydrogen storage materials at room temperature to liquid nitrogen temperatures and for high-temperature applications within nuclear energy. Graphite is used as a structural and moderator material in nuclear power reactions and in next-generation nuclear reactors. Carbon materials have also been investigated for hydrogen storage at very cold temperatures. Carbides have been studied for hydrogen barriers and recently other forms of carbon have been proposed, i.e., diamond-like carbon (DLC), graphene, metal-graphene and graphene oxide which will be discussed below. 

#### 2.3.1. Diamond-Like Carbon (DLC)

In 2017, Tamura reported the use of Diamond-Like Carbon (DLC) as an amorphous coating on 316L stainless steel with much lower hydrogen permeation resistance at 573–773 K (200–500 °C) relative to stainless steel and nitride coatings of ZrN, TiAlN, AlCrN, CrN [45]. The coatings that were pre-absorbed with hydrogen showed even lower permeability due to hydrogen being trapped and hence reducing diffusion through the coating. 

DLC is an amorphous coating with a mixture of diamond structure (sp^3^) and graphite structure (sp^2^). DLC coatings have superior properties such as high hardness, high resistance to abrasion, low friction and high insulation. DLC coatings have a wide range of applications, but tests on hydrogen barrier functions are quite limited.

Tamura showed that *DLC + buffer layers* has even better permeation resistance [45]. The buffer layer consists of two layers on the 316L stainless steel substrate; a metallic Cr coating coated with CrN with DLC coated on top of the buffer layers. The combination of the buffer layer and the DLC coating [DLC (20% H_2_)] had the lowest hydrogen permeation rate. Compared to stainless steel, the hydrogen permeation rate was reduced at least 1000 times. 

#### 2.3.2. Graphene and Graphite

Graphene has been explored for hydrogen barrier application to prevent embrittlement and oxidation of metals in industry and nuclear reactor applications [46,47,48]. It has also been investigated for hydrogen storage, typically at high pressures and very low temperatures [49]. Graphene is a monolayer of carbon atoms bound in a hexagonal honeycomb lattice forming planes of sp^2^-bonded atoms. Layers of graphene stacked on top of each other form graphite. Graphene is the thinnest material known at one atom thick, and about 200 times stronger than steel. Furthermore, graphene is a conductor of heat and electricity. It is a diverse material and can be combined with other elements (including gases and metals) to produce different materials with various superior properties. Producing high quality materials is however a challenge. Dozens of companies around the world are producing different types and grades of graphene materials, ranging from high quality single-layer graphene synthesized using a CVD-based process to graphene flakes produced from graphite in large volumes.

A paper in Nature Communications by Su et al. in 2014 [50] showed that chemically reduced graphite oxide (GO) films exhibit exceptional barrier properties with respect to all tested gases, liquids, salts and acids, with no detectable permeation. The GO films can be considered as thin graphitic linings and graphite is one of the most stable and chemically inert materials. Su et al. believes that many applications are possible when a barrier against moisture, oxygen and other gases and liquids is required. However, they do not mention hydrogen or mechanical strength and further exploration is needed.

Kim et al. [51] developed in 2016 ultra-high strength V-graphene nanolayers and the He+ irradiation study revealed excellent radiation tolerance. Interestingly, they found that inclusion of graphene in the form of V-graphene nanolayers can result in a high strength material, but the graphene can also self-heal the crystalline defects that are introduced during irradiation. Nanocrystalline V-graphene nanolayers composites were synthesized by first depositing V-monolayers with RF sputtering and then graphene was fabricated using CVD. Hydrogen permeation of metal-graphene nanolayers have not been explored. 

Yang et al. [52] investigated in 2020 GO-Al_2_O_3_ for tritium permeation barrier. The composite coating was prepared by sol-gel, creating nanosheet layers that according to their deuterium permeability tests blocked diffusion nine times higher (PRF = 9) compared to Al_2_O_3_ coating at 500 °C. Furthermore, the toughness of the GO-Al_2_O_3_ coating was enhanced 40%. 

The performance and lifetime of nuclear graphite are closely related to the irradiation environment and are affected by the specifics of the graphite: manufacturing process, graphitization temperature, composition, etc. Most graphite programs are researching unirradiated and irradiation material property performance, with a focus on irradiation creep behavior as the life-limiting mechanism for graphitic components. Irradiation damage from fast neutrons creates lattice defects leading to changes in physical and mechanical properties and the accumulation of stresses. Atsumi et al. [53] studied impact of neutron irradiation on hydrogen retention in graphite and showed that the amount of retained hydrogen increased by 100 times and the absorption rate decreased to one seventieth compared to unirradiated samples. Hydrogen trapping sites can be created by neutron irradiation which reduces hydrogen diffusivity. 

Carbon in the form of isotropic graphite is used in fluoride salt-cooled high-temperature reactors (FHRs) and thermal-spectrum Molten Salt Reactors (MSRs) as a neutron moderator. The current status of tritium control and capture is that limited data indicate it is feasible to maintain very low levels of tritium in a salt-cooled reactor using carbon. Forsberg et al. published a review in 2017 of tritium control and capture in salt-cooled fission and fusion reactors: status, challenges, and path forward [25].

#### 2.3.3. Carbides

Carbides have been studied for hydrogen permeation barriers, mainly TiC and SiC. TiC is difficult to deposit with CVD and therefore TiC-TiN has been applied obtaining 10 times higher permeation rate factor (PRF) than TiC alone [54]. Nemanič summarized PRFs for selected carbides, nitrides and oxides in a recent article from 2019 [54]. 

Tamura et al. [34] recently studied TiC along with Al_2_O_3_ and TiN, using the radio-frequency (RF) ion plating method to form films on type 316L austenitic stainless steel substrates. The hydrogen permeability decreased by at least two orders of magnitude in all test specimens after the Type SUS316L substrates were coated. Tamura et al. observed that the microstructure had an impact on hydrogen permeability.

Silicon carbide (SiC), Carborundum, is one of the proposed candidates to protect or replace zirconium fuel claddings due to higher melting temperature, better corrosion resistance at high temperatures and similar thermal neutron capture cross-section compared to zirconium. SiC coatings demonstrate high hardness, high thermal conductivity and good oxidation resistance at high temperatures. Silicon carbide is an excellent hydrogen permeation barrier. A trade-off study by INL [55] in 2012 highlights that irradiation studies have been performed and SiC was shown stable; SiC retain strength during LOCA up to at least 1300 °C. 

Although SiC is very promising, the industry must still develop a robust and commercially viable end plug joining process for final fuel rod fabrication, as current industry methods for welding end plugs on Zirconium rods will not apply to SiC composite ceramics.

Recently, nuclear fuel vendors and others have focused on cladding options and SiC/SiC composite ceramics, which provide most of the enhanced accident-tolerant characteristics. SiC is one of the hardest substances known and is widely used in wheels for cutting stone. SiC composites are structures made up of SiC fibers and SiC monolith pieces that resist the shattering experienced by most ceramics.

Kashkarov et al. [56] coated SiC as a hydrogen barrier on Zr-1Nb. Amorphous SiC coating of 1.5-µm thickness was deposited on Zr-1Nb alloy substrate by direct current (DC) magnetron sputtering of composite cathode. Hydrogen absorption decreased 8 times due to low hydrogen permeability of the coating. Hydrogenation tests show that SiC coating provides protective properties against hydrogen permeation in the investigated temperature range of 350–450 °C.

In 2020, Wang et al. [57] published a new important finding in Nature Materials on radiation-induced temperature dependent segregation in a ceramic which is different from that shown in metallic systems. Irradiation of silicon carbide at 300 °C leads to carbon enrichment near grain boundaries (GB), whereas the enrichment diminishes for irradiation at 600 °C. Wang et al. point out that radiation-induced segregation (RIS) is one of the most dramatic changes that can take place in GBs under irradiation or ion implantation and has been observed in many metallic alloys. The segregation in SiC occurred at a much lower irradiation temperature than typical RIS in metals. An ab initio informed rate theory model was used to demonstrate that this difference is introduced by the unique defect energy landscapes present in the covalent system. It is unknown how the carbon enrichment impact hydrogen behavior in SiC, but it is well known that hydride phases grow at grain boundaries. Depending on use of SiC in a nuclear reactor, it could be important to explore this effect to avoid hydrogen trapping or permeation enhancement effects. 

In 2019, Hu et al. at ORNL [58] reported hydrogen isotope permeability of SiC-based cladding. Deuterium permeation flux was measured after exposing SiC fiber-reinforced SiC matrix ceramic composites to high heat flux neutron irradiation. They studied SiC/SiC tubes as is and as coated with CrN, Cr and TiN using cathodic arc physical vapor deposition. At <250 °C, hydrogen permeability in SiC has been measured to be <1 × 10^−21^ mol H_2_m^−1^s^−1^MPa^−1/2^ [59]. Hu et al. measured deuterium leak rates at 300–500 °C as a function of deuterium pressure. A low heat flux resulted in deuterium leak rates down to 10^−11^ atm-cc/s. Higher heat flux resulted in deuterium lek rates in the range of 10^−6^ to 10^−9^ atm-cc/s. Cr-coated SiC/SiC showed lower deuterium leak rates of 10^−12^ atm-cc/s at low heat flux and lower for CrN-coated SiC/SiC. This is their first-generation coatings and they expect that future generation coatings will show better performance. 

In 2019, Terrani et al. at ORNL [60], demonstrated a new promising method to produce crystalline, high-purity SiC coatings using 3D binder jet printing. They claim that their advanced manufacturing method give complete freedom in geometric complexity which opens use of SiC on surfaces that previously were difficult to coat with other methods. They did not report hydrogen permeability which would be of interest to measure in the future to compare with previous permeability measurements of SiC.

### 2.4. MAX-Phases and MXenes

MAX phases can also provide excellent hydrogen barrier properties depending on environment. They are a group of ternary ceramic compounds composed of: **M**: early transition metals (Sc, Ti, V, Cr, Zr, Cr, Nb, Mo); **A**: elements in the A group (mainly Al, Si, P, S, Ga, Ge, Pb); **X**: carbon or nitrogen [61]. Recently, several MAX phases have been studied for hydrogen isotope barrier or nuclear cladding materials including: Ti_3_AlC, Ti_3_SiC_2_, Ti_2_AlC and TiAlN.

This group of materials needs to be much more explored to learn hydrogen barrier behavior, mechanical strength, irradiation resistance, etc. Recently, testing in radiation has been performed. Ward et al. [62] suggest that MAX phases are not suitable for in-core nuclear applications below 350 °C. The use of Ti_3_SiC_2_ and Ti_3_AlC_2_ are good examples of the inherent problem with MAX phases in irradiating environments at low temperatures. The results have shown that similar observations to neutron and heavy ion irradiation are seen with proton irradiation of Ti-based MAX phases, namely: anisotropic changes in unit cell dimensions and decomposition of the MAX phase to TiC.

In 2018, Tang et al [63] studied Cr_2_AlC and Ti_2_AlC coated on Zircaloy-4 as accident tolerant fuel cladding. Neutron radiography was used to measure hydrogen permeability but the detection limit is in the order of magnitude of 10^−4^gm^−2^s^−2^ and the permeation rate of the coatings is lower than that. Although these two coatings appear to be reducing permeation rate, more sensitive detection system is required to quantify permeability. 

In 2020, Gröner et al. [64] measured deuterium permeation of Ti_2_AlN coated on ferritic stainless-steel substrates. They also studied pre-oxidized coatings finding that that the Ti_2_AlN coating obtained a PRF = 45 relative to stainless-steel and the pre-oxidized coating obtained a PRF = 3700. 

In 2021, Tunes et al. [65] studied radiation resistance of Cr_2_AlC thin film by submitting them to heavy ion irradiations relevant to nuclear reactors. Their test was done in situ in a TEM at 623 K (350 °C). Importantly, the Cr_2_AlC material did not undergo catastrophic failure or localized fracture in contrast to other materials such as TiFeN and CrN. 

Naguib et al. [66] reviewed in 2021 ten years of progress in development of MXenes. MXenes are a new class of two-dimensional layered materials of carbides, nitrides and carbonitrides. They are called ‘MXenes’ because they are made by etching ‘A’ layer from MAX phases and the suffix ‘ene’ is added due to the similarity to grapheneThere are currently about 150 MAX-phases and 40 MXenes. MXenes can be made by selective etching of a MAX phase. 

Shi et al. [67] reported in 2021 MXene coatings as new hydrogen permeation barriers for pipe steels. The MXene (Ti_3_C_2_T_x_) nanosheets were synthesized and coated on X70 pipe steel using spin-coating in a colloidal suspension. Shi et al. measured hydrogen permeability using an electrochemical method and also assessed hydrogen embrittlement and corrosion resistance. The calculated permeability is two times lower for coated pipe compared to uncoated pipe. The barrier mechanism is due to the multilayered structures, and they discuss how difficult it is to break Ti-C bonds and form C-H bonds when hydrogen atoms diffuse. 

### 2.5. Metals

#### 2.5.1. Chromium

Chromium coatings have been much investigated recently and is a leading candidate in the nuclear industry. Framatome (formerly AREVA NP) and Westinghouse are actively developing several enhanced accident tolerant fuel claddings including Cr-coated zircaloy and SiC/SiC composite sandwich cladding. A paper by Bischoff et al. [68] focused on developing Cr-coated M5 cladding using a physical vapor deposition (PVD) prototype machine to coat full-length cladding tubes. Their characterization confirmed that Cr-coated M5 can provide significant benefits in normal operating conditions in terms of susceptibility to corrosion in harsh environments such as high lithiated water chemistry and in terms of wear resistance.

Cr is a better high-temperature oxidation resistant than Zr-4 alloy. A recent paper by Sevecek et al. [69] developed a cold spray Cr coated Zirconium-4 cladding. The coating is more nonuniform compared to PVD, but cold spray has a high deposition rate and is suitable for industry production. The samples were oxidized in high-temperature steam at 1200 °C and efficiently prevented oxidation.

#### 2.5.2. Tungsten, Gold, Silver, Platinum, etc.

A review of hydrogen permeability data of solid metals in Table 1 shows that there are a few pure metals with very low permeability [70]. Some of the above discussed carbides, nitrides and oxides have lower permeability than the metals [54]. 

REB Research and Consulting published a graph [71] in 1996 that displays hydrogen permeability vs. inversed temperature for numerous metals, including Ag, Au, Pt, Cu. Gold has the lowest permeability followed by silver, aluminum, platinum, cupper, iron and palladium. 

### 2.6. FeCrAl Alloys

FeCrAl alloys have been investigated during the past 50 years for cladding and hydrogen permeation barrier applications in nuclear industry; for nuclear power applications including accident tolerant fuel (ATF) cladding, structural components for fast fission reactors, and as first wall and blanket structures for fusion reactors. FeCrAl alloys are under consideration for these applications due to their inherent corrosion resistance, stress corrosion cracking resistance, radiation-induced swelling resistance, and high temperature oxidation resistance and hydrogen pick-up resistance. Handbook on the Material Properties of FeCrAl Alloys for Nuclear Power Production Applications [72] was published in 2018 by Field et al. (ORNL report) and Garud et al. [73] reviewed in 2022 hydrogen isotope permeation in clean and unoxidized FeCrAl alloys. Hydrogen permeation rates in ferritic steels are higher and hydrogen solubility is lower than in austenitic steels. The tritium permeation rates in FeCrAl alloys are between those in austenitic stainless steels and in ferritic FeCr steels.

Field et al. [72] report that Hydrogen/Tritium permeation has been measured at 200–700 °C for different FeCrAl alloys to determine permeability and possible mitigation strategies. In general, the higher Cr content FeCrAl alloys show lower permeability than the lower Cr content alloys. Additionally, a significant difference in the permeability between non-oxidized and oxidized specimens is apparent. Oxidized specimens with Al_2_O_3_ layer have been shown to have nearly one order of magnitude lower permeability than typical bare FeCrAl alloys. 

#### Molybdenum Diffusion Barrier Layer with FeCrAl

Yeom et al. [74] showed that Mo diffusion barrier layer between FeCrAl coating and Zr-alloy substrate is essential for accident tolerant fuel applications. To enhance oxidation resistance at high temperatures (>1000 °C) in the event of loss of coolant accident (LOCA), the cold spray process was investigated for the deposition of FeCrAl coatings on optimized ZIRLO™ fuel cladding tubes. An optimized FeCrAl-Mo coating was shown to effectively protect the Zr-alloy substrate from oxidation. Hydrogen barrier properties are however unknown and would be of interest to explore. 

## 3. Summary of Advanced Coatings for Hydrogen Barrier Applications and Future Prospects

**Figure 3:**
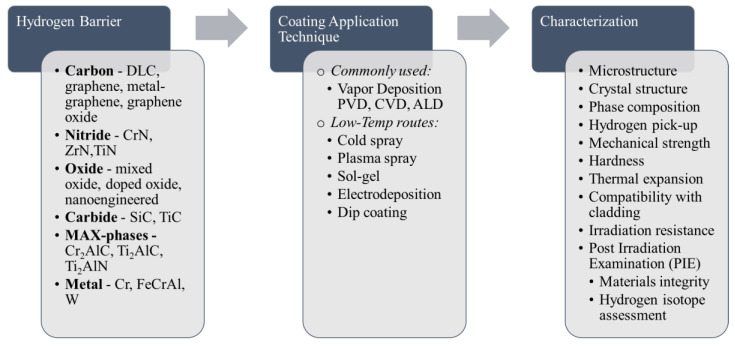
Summary of Advanced Hydrogen Barriers, Application Techniques and Characterization.

We have in this review of recent literature (most papers were published within the past 5–7 years) identified promising hydrogen isotope barrier coatings and knowledge gaps to further advance the state of the art. Below is a list of hydrogen barriers that recently have been in focus for development for various applications. Figure 3 shows a summary of recently developed hydrogen barriers.

- Carbon: diamond like carbon (DLC); graphene, metal-graphene, graphene oxide.
- Carbides: SiC, SiC/SiC, TiC, TiAlC layered phase.
- MAX-phases and MXenes.
- Metals: Cr, FeCrAl alloys, FeCrAl + Mo diffusion barrier.
- Nitrides: CrN, ZrN, TiN.
- Oxides: dopant + ZrO_2_; bipolar oxide Al_2_O_3_/Cr_2_O_3_, nano-Al_2_O_3._

### Identified Knowledge Gaps—Future Research Prospects

**Figure 4:**
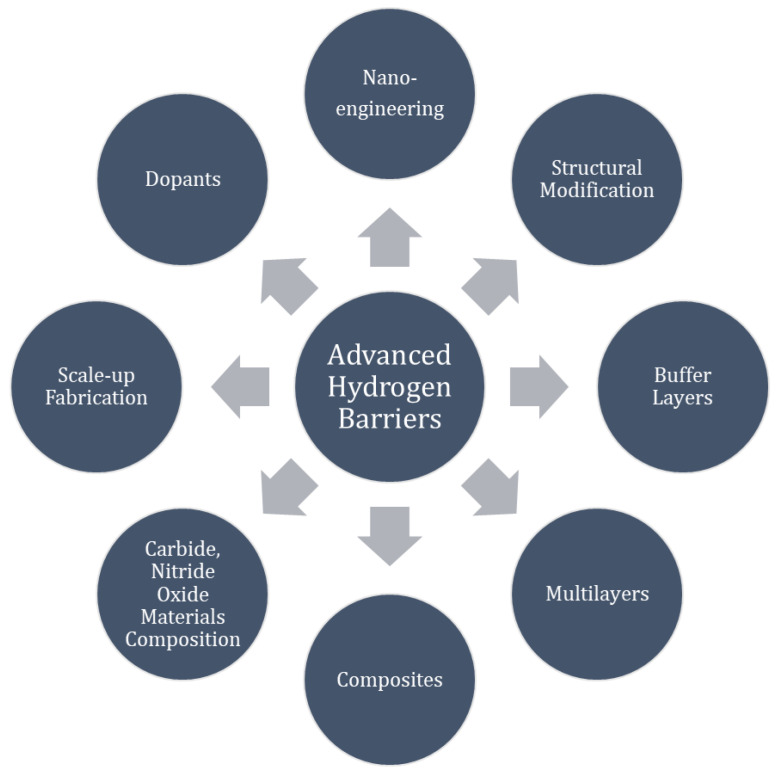
Advanced Hydrogen Barriers; key materials and material engineering strategies.

Depending on the environment applicable to the application, further studies are needed to fill knowledge gaps. The following list—in no particular order—represents knowledge gaps in the application of advanced hydrogen barriers to reduce hydrogen absorption and permeation and identifies plenty future research prospects. Figure 4 illustrates key materials and materials modification strategies to improve properties.

- Perform hydrogen absorption and permeation measurements in environments relevant to the application.
- Correlate permeation rate with hydrogen trapping, solubility, diffusivity, microstructure, phase formation, grain boundaries and fabrication procedure to learn how to tailor materials to reduce hydrogen permeability.
- Utilize nanoscience, structural modifications and recoverable Nano Lattices to reduce hydrogen permeability.
- Advance structurally modified oxide materials; dopants; nano-engineering; nanoceramics.
- Explore combinations of metals and metal alloys with diffusion barrier.
- Exploration of the impact of dopants and alloying elements.
- Explore bipolar oxides.
- Explore diamond like carbon with buffer layer of CrN, etc.
  o Micro or nanostructure engineering.
  o Key is structural control.
- Study of microstructure related to fabrication method to identify improved performance.
- Exploration of multilayer structures with hybrid architecture for evaluation with respect to mechanical strength and manufacturing feasibility.
- Identification of cost-effective, low-temperature preparation method to avoid HAZ.
  o For example; using cold spray, sol-gel routes, chemical spray pyrolysis or nitrates and triblock copolymers for wet chemistry routes.
- Develop new feasible scale-up fabrication techniques of low cost.
- Study of radiation resistance in nuclear reactor applications.
  o Structural modifications to improve radiation resistance in a nuclear reactor.

## Figures and Tables

**Table 1:** Hydrogen permeability of selected metals at 500 °C [70].

Metal	Permeability mol H_2_/m/s/Pa^0.5^
Vanadium	2.9 × 10^−8^
Niobium	7.5 × 10^−9^
Titanium	7.5 × 10^−9^
Nickel	1.2 × 10^−10^
Ferritic Steels	3 × 10^−11^
Austenitic Steels	0.7–1.2 × 10^−11^
Molybdenum	1.2 × 10^−11^
Tungsten	4.3 × 10^−15^

## Data Availability

Not applicable.
